# Supplementary material for: Are carbon emissions trading and green financial instruments synergistic? -Comprehensive quantitative research based on content analysis
Source: PLoS One. 2024 Mar 7;19(3):e0298601. doi: 10.1371/journal.pone.0298601 (PMC10919601; doi:10.1371/journal.pone.0298601)
Supplement: S1 Data — (ZIP) [file pone.0298601.s001.zip › Data/地级市Z_F工作报告.zip!/╣π╬≈/╬α╓▌╩╨/└┤╫╘╣1⁄2╓┌║┼í╛┬φ┐╦╩2╛▌═°í┐_2010.docx]

各位代表：

　　现在，我代表市人民政府向大会作政府工作报告，请予审议，并请市政协委员和其他列席会议的同志提出意见。

　　2009年工作回顾

　　2009年，是我市进入新世纪以来经济发展压力最大、任务最重、困难最多的一年，也是全市干部群众凝聚力最强、经济发展速度最快、人民群众得实惠最多的一年。面对复杂严峻的经济发展形势，我们在自治区党委、政府和市委的正确领导下，在市人大、市政协的监督支持下，深入贯彻落实科学发展观，与全市人民一道，同心同德，众志成城，沉着应对国际金融危机，保持了经济快速发展势头，多项经济指标增速排在全区前列，主要经济指标提前一年完成“十一五”规划目标，圆满完成了市十二届人大五次会议确定的各项任务。

　　经济保持快速增长 预计全市实现地区生产总值458亿元，增长17.5%，增速排全区第2位，创16年来最高增幅；完成财政收入40.06亿元，增长23.6%，增速排全区第3位；完成全社会固定资产投资330亿元，增长66.4%，增速排全区第2位，年增量首次突破100亿元，人均固定资产投资突破1万元，投资拉动GDP增长12个百分点；城镇居民人均可支配收入14600元，增长10%；农民人均纯收入4240元，增长10%。综合经济实力明显提升，经济总量在全区排位上升至第5位，规模以上工业增加值上升至第4位。

　　工业经济逆势上扬 全年完成工业总产值690亿元，增长18.5%，人均工业产值突破2万元；完成工业增加值232.4亿元，增长23.5%。规模以上工业企业完成工业增加值165.2亿元，增长30%，盈亏相抵后实现利润总额13亿元，增长30%。全市完成技改投资89.6亿元，增长172.9%，完成自治区下达任务的135.8%，增速创历史最高水平。市本级36家重点工业企业完成工业总产值96.9亿元，增长36.9%；主营收入、盈亏相抵后实现利润、税收入库分别增长42.6%、88.8%、33%。中恒集团公司、神冠蛋白肠衣公司、丰业集团公司、梧州供电局年纳税额超亿元。神冠控股（集团）有限公司在香港成功上市。园区经济不断壮大，园区工业总产值达115亿元，增长21.1%。新中陶、30万吨再生铜等重大工业项目开工建设。积极推进节能减排工作，全面完成年度化学需氧量和二氧化硫排放总量削减任务。

　　农村经济稳步发展 全年农业总产值达117.6亿元，增长7.1%，增速排全区第2位。粮食播种面积236.1万亩，粮食产量84.4万吨，增长6.5%，超额完成自治区下达的任务。全面落实粮食综合直补、水稻良种补贴等支农惠农政策，全年发放支农惠农补贴资金2.03亿元。优质稻、亚热带名优水果、速丰林、木本天然香料、三黄鸡、瘦肉型猪等专业化生产基地初具规模，建成了全国最大的六堡茶生产加工基地和全区最大的砂糖橘种植销售基地、肉兔养殖加工基地、中草药种植基地。加快农村改革步伐，推进农村土地承包经营权流转规范化管理和集体林权制度改革试点工作，有效地促进了农业优势产业向专业化、规模化、基地化发展。健全重大动物疫病防控体系，不断完善预警预报机制。扎实推进以水利设施、乡村道路为重点的农村基础设施建设，全市实施各种水利工程项目239项，完成投资4.26亿元。县域经济发展取得重大突破，岑溪市、藤县获广西科学发展十佳县（市）称号，苍梧县获广西科学发展进步县称号。

　　交通建设实现突破 西江亿吨黄金水道和西江经济带建设加快推进。贵港至梧州Ⅱ级航道整治主体工程完工通航，长洲水利枢纽三四线船闸开工建设，梧州港赤水圩作业区五号泊位主体工程、李家庄码头二期工程完工。梧州港货物吞吐量达1130万吨，集装箱15.3万标箱。南广铁路建设全面展开，洛湛铁路开通运营，结束了梧州不通火车的历史。桂梧高速公路全线贯通，贵港至梧州、岑溪至水汶高速公路开工建设。苍梧龙圩至藤县潭东、至梧州进口再生资源加工园区二级公路改一级公路项目开工建设，梧封一级公路、苍梧环城一级公路路面改造工程完工。全年交通项目完成固定资产投资72.4亿元，为我市历年来交通基础设施投资最大的一年。

　　城镇建设步伐加快 全市城市建设项目完成投资20亿元。城市路网、电网、水网改造工程加快建设。红岭片、三龙片、南岸片区开发前期工作有序推进。西江三桥、文化中心改造、新兴路综合整治、榜山休闲公园、市县污水垃圾处理等项目加快推进。创建国家森林城市申请获国家林业局批准。全年新增绿地13.8万平方米，改造绿地18.4万平方米。城乡风貌改造工程、城乡清洁工程、打击市区非法占地违章建筑工作取得新进展。各县（市）和各建制镇城镇规划建设力度不断加大，城镇化率达40.1%，提高0.6个百分点。

　　开放合作不断深化 全年实现外贸进出口总额5. 58亿美元，增长9.8%，其中出口3.88亿美元，增长7.3%。成功在广州、佛山等地举办招商推介会，政府自建工业厂房全面完成招商任务，全市招商引资实际到位资金201.3亿元，完成目标任务的129%，增长54.9%。成功举办第六届梧州国际宝石节，并成为中国—东盟博览会系列活动之一。西江沿线8市共同签署了发展循环经济、建设生态西江的《梧州宣言》，合作不断深化。成功承办第三届中国中医药发展大会，有力推动了我市中医药产业的发展，打出了“医药梧州、医疗梧州”的新名片。

　　消费市场繁荣活跃 全年社会消费品零售总额达171.3亿元，增长19.5%。积极推进“家电下乡” 、“万村千乡”和“双百市场”等工程，全市新建各类农家店270个，累计发放家电下乡产品补贴资金1143.1万元，发放汽车、摩托车下乡补贴资金2617.3万元，惠及农户3.5万多户。全年累计接待游客558.6万人次，旅游总收入34.2亿元，分别增长14.8%和23.9%。中恒国际医药商贸物流基地、易达物流等项目实现开竣工。

　　民生工程扎实推进 全市返乡农民工12.7万人，实现就业11.5万人，就业率达91.3%；全市城镇登记失业率为4.04%，低于目标控制数0.46个百分点。新型农村合作医疗保险工作稳步推进，全市参合农民224.16万人，参合率达90.02%，为参合农民报销医药费1.98亿元。城乡居民最低生活保障制度进一步完善，城市低保标准提高到每人每月255元，低保对象月人均补助由130元提高到150元；农村低保标准提高到每人每年1196元，低保对象月人均补助由35元提高到50元。继续健全城乡医疗救助制度，全额资助城乡低保户、农村五保户参加城镇居民基本医疗保险和新型农村合作医疗保险。为民办的12件实事全部完成。平民冲地质灾害综合整治工程进展顺利。认真落实大中型水库移民后扶政策，库区移民生产生活条件不断改善。

　　社会大局和谐稳定 深入开展重大安全隐患排查和专项整治，不断加强安全生产责任的落实，各类事故发生起数大幅下降。大力加强食品药品监督管理，保障了食品药品安全。完善应急预案，有效预防和处置各种突发公共事件。认真做好新时期信访和人民调解工作，加强社会矛盾的排查化解，确保了新中国成立60周年、第六届中国—东盟博览会、第六届梧州国际宝石节等重大节庆活动期间的社会稳定。深入开展社会治安综合治理，严厉打击违法犯罪行为，扎实推进平安梧州建设，为发展创造了和谐稳定的环境。

　　社会事业全面进步 人口和计划生育工作扎实推进，人口出生率、自然增长率、出生政策符合率均完成自治区下达的控制目标。科技创新计划和全民科学素质行动计划顺利实施，全年新增高新技术企业3家、高新技术产品14个。加大教育、文化、卫生、体育等领域的投资力度，公共管理和公共服务水平进一步提升，全市教育支出 16.13亿元，增长 19.9%；医疗卫生支出 6.78亿元，增长77.3%；城乡社区事务支出 5.61亿元，增长118.7%。教育惠民工程扎实推进，本科和本专科上线率排名广西第一，县（市）职业教育中心建设走在全区前列。文化体育项目加快建设，市图书馆、博物馆、体育中心等项目开工建设，完成10个乡镇综合文化站和95个农村篮球场建设。重大疾病防控工作成效显著，有效控制了甲型H1N1流感暴发流行。广播电视“村村通”工程加快推进，全年投入309万元，为6838户村民安装开通了卫星电视。

　　一年来，各级政府按照为民、务实、清廉的要求，扎实开展深入学习实践科学发展观活动，加快政府职能转变，加强政府法制建设，强化行政监察、审计和工作督查，全面落实廉政建设责任制，政府自身建设迈出新步伐。国防动员、国资监管、国土资源、人事、统计、法制、民族、宗教、对台、外事、侨务、农机、供销、工商、税务、质量技术监督、海关、海事、通信、检验检疫、地震、人防、新闻出版、地方志、档案、扶贫等工作取得新进展，工会、共青团、妇女儿童、老龄、残疾人等事业取得新进步。

　　总结一年来的工作，我们在应对国际金融危机冲击，全力保增长、保民生、保稳定和保持发展良好势头的这场重大考验中，既取得了显著的经济成果，又积累了在复杂经济环境中加快发展的重要经验。我们的主要体会有：必须坚持把保持经济平稳较快发展作为经济工作的首要任务。梧州作为欠发达后发展地区，无论形势如何变化，我们都要牢牢抓住发展这个第一要务不放松；必须坚持把扩大投资需求作为经济增长的主要拉动力。梧州经济发展的阶段特征决定了当前和今后一个时期扩大投资仍是拉动经济增长最直接、最有力、最有效的途径；必须坚持把调整经济结构作为推动经济发展的根本途径。通过调整产业结构，加快转变经济发展方式，做大做强工业，加快发展现代服务业，不断培育新的经济增长点；必须坚持把“以空间换时间、以资源换产业、以存量换增量”作为加快经济发展的重要切入点；必须坚持把“抓住一个关键、确保两个稳定、实现三个突破”作为推动经济发展的重要着力点；必须坚持把保障和改善民生作为经济工作的根本出发点。做好保障和改善民生工作，是坚持以人为本、实现发展成果由人民共享的必然要求，也是扩大内需、拉动经济增长的重要动力；必须坚持把转变作风作为推动经济发展的重要保证。树立和弘扬真干实干苦干的优良作风，切实增强危机意识、忧患意识，是攻坚克难的重要法宝。在困难面前，只要我们始终保持良好的精神状态，坚定加快发展的信心，就一定能够化压力为动力，化挑战为机遇，化危机为转机，不断夺取经济社会发展的新胜利。

　　各位代表，成绩来之不易，经验弥足珍贵。这是党中央、国务院和自治区党委、政府作出一系列正确决策部署的结果，是市委正确领导、人大有力监督、政协大力支持的结果，是各级各部门和全市各族人民同心协力、顽强拼搏的结果。在此，我代表市人民政府，向辛勤工作在各条战线上的工人、农民、知识分子、干部和社会各界人士，向驻梧部队、民兵预备役部队官兵和政法干警，向人大代表、政协委员，向各民主党派、人民团体，致以崇高的敬意和衷心的感谢！

　　在肯定成绩的同时，我们也清醒地看到，我市经济增长的基础还不够稳固，发展中还存在不少困难和问题：国际金融危机的影响尚未消除，国内外经济形势不确定因素仍然存在；经济总量小、经济结构不合理、经济增长的质量和效益还不高；重大项目储备不足，前期工作推进困难，保持投资持续较快增长的压力还很大；中小企业融资难的问题依然存在，扶持企业做大做强的力度还需加大；统筹城乡发展的任务还很艰巨，农民持续增收的难度还很大；政府自身建设还需进一步加强等。对此，我们一定高度重视，认真加以解决。

　　2010年工作总体要求和目标任务

　　今年是实施“十一五”规划和“科学发展三年计划”的最后一年，也是继续应对国际金融危机确保经济平稳较快发展的关键之年。总体上看，今年的经济发展环境要好于去年，但面临的形势仍然较为复杂，对我们的影响仍然难以预料。在这种复杂形势下，我们既要看到发展面临的不利因素，更要看到加快发展的难得机遇和有利条件：国务院出台关于进一步促进广西经济社会发展的若干意见，实施新一轮西部大开发战略，自治区加快推进西江亿吨黄金水道和西江经济带建设，是我们谋取新发展、创造新业绩的坚实基础；一批规模大、起点高、带动力强的基础设施、基础产业项目正极大改善我市的发展环境和发展条件，是我们谋取新发展、创造新业绩的坚强支撑；全市上下形成的好思路、好做法、好作风、好氛围、好势头，是我们谋取新发展、创造新业绩的强大动力。

　　根据中央和自治区经济工作会议的要求和部署，结合我市实际，2010年政府工作的总体要求是：以邓小平理论和“三个代表”重要思想为指导，以科学发展观为统领，认真贯彻党的十七大和十七届三中、四中全会精神，全面落实中央经济工作会议精神和国务院关于进一步促进广西经济社会发展的若干意见，按照自治区党委郭声琨书记在梧州考察调研时的重要讲话精神，牢牢抓住西江亿吨黄金水道和西江经济带建设等重大机遇，巩固基础，深化发展，着力推动发展方式转变和经济结构调整，着力扩大内需保增长，着力提高经济增长质量和效益，着力推进改革和自主创新，着力深化对外开放和区域合作，着力保障和改善民生，保持经济快速增长的良好势头和社会和谐稳定，加快 “两城市一基地”的建设步伐。

　　2010年经济社会发展的主要预期目标是：地区生产总值增长13%，全社会固定资产投资增长30%，财政收入增长15%，社会消费品零售总额增长16%。万元生产总值能耗持平，二氧化硫排放总量控制在4.69万吨以内，化学需氧量排放总量控制在6.50万吨以内。城镇新增就业1.6万人以上、城镇登记失业率控制在4.5%以内。城镇居民人均可支配收入增长8%，农民人均纯收入增长8%。人口自然增长率控制在10％。以内，居民消费价格涨幅控制在5%左右。

　　做好今年的政府工作，必须把转变经济发展方式作为深入贯彻落实科学发展观的重要目标和战略举措，抢抓西江亿吨黄金水道和西江经济带建设这一重大战略机遇，把工业化、城镇化作为加快发展的主攻方向，把项目建设作为加快发展的主要抓手，树立追赶意识，强化责任意识，咬定发展不放松，咬住目标不动摇，咬紧牙关不松劲，努力保持经济快速发展的良好势头。

　　2010年主要工作部署

　　实现今年工作目标任务，必须顽强拼搏，狠抓落实，乘势而上。重点抓好以下10项工作：

　　一、坚定不移推进重大项目建设，保持投资对经济增长的强劲拉动

　　全力打好项目攻坚战 实施重点建设项目488个，总投资1312亿元，年内计划投资369.2亿元。其中，新开工项目268个，年内计划投资148.7亿元，力争新开工柳州至肇庆铁路梧州段、岑溪至罗定地方铁路、梧州港赤水码头铁路专线、环城高速公路、荔浦至玉林高速公路梧州段、梧州机场改造等重大项目。续建项目220个，年内计划投资220.5亿元，主要建设南广铁路、梧州至贵港高速公路、岑溪至水汶高速公路、长洲水利枢纽三四线船闸、李家庄码头三期、塘源综合码头、粮食专用码头、梧州进口再生资源加工园区、梧州陶瓷产业园区等重大项目。年内完工项目200个，其中上亿元项目102个，主要加快梧州港赤水圩作业区5个2000吨级泊位和一批110千伏变电站等项目建设，确保项目按计划竣工投产。

　　加大项目资金筹措力度 积极争取上级资金支持，努力筹措政府性资金，力争中央和自治区补助资金2.5亿元以上。加大投融资力度，依托政府投融资平台，力争新增融资15亿元。加强政银合作，加大引银入梧力度，推动建立西江经济带投资开发基金，引导金融机构加大对重点工程和优势产业的信贷支持力度，力争全年新增贷款100亿元。支持企业发行债券。大力引导社会资金投向基础设施、公用事业、社会事业等重点领域和薄弱环节，鼓励民间资本进入新能源、环保、生物医药、电子信息等产业，力争社会资金投资达到25亿元以上。

　　切实保障项目建设用地 加强土地管理，优化用地结构，优先保证基础设施、重大项目、民生工程和社会事业用地。探索农村土地市场建设，研究推进农村宅基地、农村土地承包经营权流转办法。加大闲置土地处置力度，积极盘活利用现有存量土地。严格执行用地项目的容积率、投资强度、建筑密度等控制指标，提高节约集约用地水平。加大土地开发整理力度，依法保护耕地。

　　加大项目前期工作力度 安排重点前期工作项目61个，总投资1021.5亿元。重点突破西气东输二线工程、洛湛铁路电气化、梧州至柳州高速公路、梧州至信都高速公路、梧州火电、梧州核电、西江黄金水道数字化航道等重大项目的前期工作。加大新项目研发力度，为保持投资增长后劲储备项目。进一步完善项目联合审批制度，整合项目咨询评估、审批核准、规划、用地、环保等前置审批环节的力量，提高项目备案、核准、用地预审、环评等相关审批效率。用足用好国家的各项政策，主动对接，争取更多的项目列入自治区统筹推进项目盘子。加大前期工作经费投入，保证重点项目前期工作顺利推进。

　　健全项目建设保障机制 巩固完善“五个一”的项目推进机制，坚持“四个非常”的举措，切实解决项目审批、征地、搬迁、资金、环评等项目建设实际问题，加强对重大项目工程招投标、进度、质量、安全、资金、采购等环节的督促检查，营造相互支持、协同作战、共同推进项目的良好氛围，努力完成全年投资目标任务。

　　二、坚定不移推进产业结构调整，做大做强做优工业

　　加快工业项目建设 坚持以重大工业项目支撑优势产业。加快30万吨再生铜、神冠蛋白8亿米肠衣生产线、梧州松脂、联溢化工等72个续建项目建设。加快推进林浆纸一体化、燃料乙醇、30万吨再生铝等重大项目的前期工作，力争今年开工建设。建设再生资源利用、陶瓷、有色金属精深加工、医药、造船及船用机械等一批重大项目，为百亿元、千亿元产业和特色产业发展奠定基础，振兴梧州老工业基地。

　　加大技改投入力度 加强工业技术改造，提高工业生产的自动化、信息化水平。围绕优势产业、龙头企业科学谋划，组织实施164个技改项目，力争全年技改投资突破120亿元。加大对重点技改项目扶持力度，对投资在1000万元以上的项目进行重点跟踪服务。积极构建企业融资平台，拓宽融资渠道，通过争取上级资金支持、设立技改专项扶持资金和金融信贷等方式，筹措项目建设资金，努力增加资金投入，缓解工业企业技改资金短缺的困难。积极发挥科技孵化园作用，支持企业与高等院校、科研院所合作建立研发中心，开发新产品、新技术，促进科技成果转化和产业化，提高企业核心竞争力。

　　加快产业园区发展 坚持把园区作为发展工业的主战场，进一步完善园区的基础设施和配套设施，推动新增工业项目向园区集聚、技改项目“退城进园”，促进企业集聚，形成产业集群。以梧州工业园区为主体，打造医药、食品产业集群；以梧州高新技术产业园区为主体，打造高新技术产业集群；以梧州进口再生资源加工园区为主体，打造再生资源加工利用产业集群；以梧州陶瓷产业园区为主体，打造陶瓷产业集群；以长洲特种钢产业园区为主体，打造特种钢产业集群；以塘源化工产业园区为主体，打造化工产业集群。推进县域特色产业集中区建设。力争全市园区工业总产值达225亿元以上，税收达10亿元以上。

　　提高服务企业水平 深化“服务企业年”活动，切实为企业提供优质服务，促进企业做大做强，力争产值超10亿元企业达7家以上，税收超亿元企业达7家以上。大力支持中恒集团、神冠蛋白肠衣公司、丰业集团公司等企业进一步做大做强。大力实施质量兴梧战略，创建高新技术创业服务中心。大力实施品牌战略，推动企业创名牌，再创一批国家级、自治区级品牌和著名、驰名商标，推动日成林化公司、天鹅蓄电池公司、华虹股份公司、亿丰新宇公司等企业上市。大力发展非公有制经济，促进非公有制经济快速发展。加强行业协会和商会管理，引导行业协会、商会规范发展。

　　三、坚定不移加强“三农”工作，促进农业增产农民增收

　　稳定发展粮食生产 进一步落实粮食直补、农资综合直补、水稻良种补贴、农机购置补贴等强农惠农政策。实行粮食生产行政首长负责制，重点实施60万亩超级稻及综合增产配套技术推广项目，稳定粮食播种面积，提高粮食单产，确保粮食播种面积达237万亩，总产量达85万吨，增长0.7%。

　　加快现代农业发展 加快发展砂糖橘、中草药、六堡茶、蚕桑、速丰林、软枝油茶等优势种植业，推进千亩中草药良种繁育和标准化示范基地、千亩六堡茶标准化茶园和六堡茶集中加工区建设。加强砂糖橘黄龙病防控，加快产地市场和采后加工处理生产线建设。加快发展生猪、三黄鸡、养兔、网箱养鱼、养蜂等特色养殖业，推进优质畜禽水产品养殖规模化。大力发展林下经济，提高林地使用效益。努力办好第三届广西（梧州）春茶节暨六堡茶博览交易会。

　　加强农业农村基础设施建设 继续开展县乡公路建设大会战，推进县、乡、村客运站和便民候车亭建设。加快病险水库除险加固。实施农村饮水安全工程，新增解决15万以上农村人口饮水安全问题。抓好以治旱设施建设和水毁农田水利设施修复为重点的一批农田水利设施项目建设。加强水资源保护和水土保持工作。加强农村户用沼气池建设，提高农村沼气的入户率。大力开展农村危房改造，进一步改善农村居住环境。加大农机推广力度，提高农业生产效率。

　　深化农业农村改革 全面推进集体林权制度改革，确保完成自治区下达的林改勘界面积731万亩，林改发证面积589.3万亩，其中藤县基本完成集体林权制度改革任务。积极推行“农事村办”和村级“四议两公开”工作。积极推进农村金融综合改革，扩大农户小额贷款发放面。建立10个县（市、区）级新农村建设示范点，其中5个点列为市级示范点，力争新农村建设在乡村旅游、优势产业发展上取得新突破。

　　四、坚定不移统筹城乡发展，加快城镇化进程

　　加快中心城市发展 坚持新城区开发与老城区改造并重，内涵提升与外延扩展相结合，加快建设整体功能完善、生态环境优良、现代气息浓郁的中心城市，实现城市规模大幅扩容，城市承载和辐射功能显著增强，城市形象品位明显提升。加快红岭三龙新城建设，重点推进路网工程、玫瑰湖景区、景观大道等项目建设，力争基本建成红岭三龙主干道路网。加快启动南岸新区开发，力争开工建设西江四桥，逐步把圣文园、八宝塘、下小河和长洲水利枢纽库区整体打造成为高尚休闲度假旅游区。加大旧城改造力度，开工建设桂江一桥加固改造工程，加快平民冲、冰泉冲、石鼓冲的地质灾害综合整治，推进白垢村和河东片的危旧房改造。

　　加强县城和小城镇建设 加快把岑溪市建设成为副中心城市，把藤县建设成为中等城市，把蒙山县建设成为特色小城市，实现市区与苍梧县城同城化。加快重点小城镇建设，把新地、大业、塘步、太平等多个区位优势明显、产业基础较好、城镇化水平相对较高的乡镇建设成为广西经济强镇。抓好以梧州工业园区、梧州港（赤水）工业区、进口再生资源加工园区、陶瓷产业园区、塘源化工产业园区为依托的小城镇规划，配套提升道路、管网、社区服务等设施，提升其承接和带动功能。加大推进土地流转、农村户籍制度改革力度，切实放宽户籍限制，统筹引导农业人口到城镇就业和定居，鼓励有稳定职业和收入的农民工及其子女转为城镇户口，纳入城镇社会公共服务体系。

　　大力塑造城乡特色风貌 扎实开展国家森林城市创建活动，营造“大种树、人人种、年年种”的氛围，突出抓好城市建成区的绿带、绿道、绿岛建设，增加城市绿量，打造西堤路、火车站站前大道、红岭一二号路等几条城市主干道绿化精品；加强森林生态网络建设，重点抓好高速公路、铁路、国道沿线、西江黄金水道沿岸绿色通道建设以及城镇村屯绿化。大规模开展城乡风貌改造，按照“蓝天白云、青山绿树、碧水红瓦”的要求，城市风貌改造重点是改造“清水墙”，杜绝新的“竹筒房”，广泛开展小街小巷改造和小景点、小绿地、小活动场所建设；农村风貌改造重点是做好“路树墙、沟池场、文卫网，外加一栋小红房”的文章。加强农村建房规划，开展整村连片治理，改变农村“有新房无新村，有新村无新貌”的现状。深入开展城乡清洁工程，继续加大“五乱”治理力度，树立清洁、整齐、美观的城乡新形象。

　　促进房地产业稳定发展 进一步加强房地产市场调控，完善住房消费的税收、信贷政策，支持居民自住和改善型住房消费。增加中小户型、中低价位普通商品房用地供应，鼓励县城和重点镇发展房地产业，规范发展住房二手市场，盘活住房租赁市场。加强市场监管，维护房地产市场秩序。加大廉租住房、经济适用住房建设力度，推进城镇危旧房改造，开展经济租赁住房、限价房建设试点，切实解决好特殊群体和城镇中低收入家庭住房困难问题。

　　五、坚定不移扩大开放合作，推进西江经济带建设

　　完善发展规划 围绕西江经济带建设的战略部署，调整完善城市总体规划、产业发展规划、港口码头规划、综合交通运输规划等专项规划，以规划引导西江黄金水道科学合理开发，促进产业沿江集聚，重点布局内河临港工业、现代物流业、现代农业和旅游业，逐步形成分工明确、优势明显、协作配套的产业带，推进全市50%的人口、70%的产业向西江沿线集聚，实现城镇与产业的集聚发展，推动西江经济带形成。

　　深化交通发展 围绕建设国内区域性综合交通运输枢纽，加快构建公路、铁路、水运、航空协调发展的现代综合交通体系。完善综合运输体系交通布局，探索综合运输枢纽的建设和运营模式，统筹各种运输方式的建设，重视城市交通运输与其他运输方式的对接，努力实现多种运输方式的“无缝衔接”和“零距离”换乘。全力推动交通重点项目建设进程，尽快形成交通体系主骨架。加强国省干线公路的养护工作，加快县乡公路建设步伐，提高路网通行能力和通达深度。积极开通梧州机场新航线，确保航空客货运量稳定。

　　承接产业转移 开展“招商引资年”活动，整合各方力量，统筹推进全市招商引资工作。主攻粤港澳，拓展长三角，围绕产业、依托资源招大引强，力争引进一批大企业、大项目。依托特色产业园区和优势企业，引进上下游配套企业，拉长产业链条，形成特色产业集群。优化投资环境，推进招商引资项目大兑现，确保招商项目合同履约率超过95%、开竣工率超过60%、资金到位率超过40%。

　　加强区域合作 主动融入珠三角，主动对接北部湾，推动资源共享、产业配套、交通对接、环保同治，把西江黄金水道建设成为生态、高效的“绿色运输大通道”。办好第七届梧州国际宝石节。充分利用中国—东盟自由贸易区平台，鼓励企业到东盟建立产品加工基地、农业综合开发加工基地、矿产资源综合开发基地。拓展与长三角地区、环渤海地区以及台湾地区的经贸合作。加快沿江电子口岸平台建设，规划设立出口加工区，提高口岸对外开放水平。进一步发展对外贸易，力争全市外贸进出口总额增长10%。

　　六、坚定不移扩大消费需求，加快现代服务业发展

　　发展商贸物流业 围绕西江黄金水道建设，把发展现代物流业作为调整优化产业结构的突破口，着力培育扶持一批物流龙头企业，建设一批专业物流基地，积极引进国内外知名商贸物流企业，重点加快中恒国际医药商贸物流基地等现代商贸物流项目建设。加快城乡商业网点规划建设，重点改造和培育两广综合市场、建材专业市场、装饰材料专业市场等一批大型批发市场，增强市场辐射功能和拉动效应。深入推进“万村千乡”、“双百市场”和“新农村现代物流”工程建设。

　　加快发展旅游业 围绕旅游目的地和游客集散地建设，统筹规划，加快建设梦都美生态园、茶花主题生态园、体育雕塑主题生态园。提升骑楼博物城、龙母庙、历史文化长廊、石表山风景区质量，加快四恩寺、天龙顶、李济深故居、太平天国永安州活动旧址、袁崇焕故居等十大旅游景点景区建设。加快江滨、国龙等五星级酒店和会所建设，鼓励和扶持现有宾馆酒店改造升级，全面推进旅游行业标准化体系建设，提高接待能力和接待水平。发展休闲度假旅游，实施乡村旅游富民工程。加大 “梧州三宝”即人工宝石、六堡茶、龟苓膏的宣传推介力度，推动旅游产品的多样化、特色化、品牌化。深化与南宁、桂林旅游区和粤港澳旅游区的区域旅游合作。力争全市旅游总收入达38亿元，接待游客总人数达620万人次。

　　大力发展文化产业 筹建文化传媒有限公司，积极发展文化创意、演艺娱乐、广告会展、出版发行、网络数字等产业。支持文化休闲健身、艺术培训等社会文化产业的发展。推进文化与旅游相结合，挖掘岭南文化的深刻内涵，逐步打造历史文化、宗教文化、龙母文化、骑楼文化、粤剧文化等具有地方特色的文化品牌。加快图书馆、博物馆等基础设施建设，创建历史文化名城。建设文化市场体系，拓展文化消费市场，创新文化产品生产和服务，培育新的文化消费热点。进一步加强文化市场监管。

　　七、坚定不移加强环境保护，推进生态文明建设

　　大力发展生态经济 良好的生态环境，是梧州发展的生命线和核心竞争力。积极发展低碳经济，在生产生活领域大力推广清洁能源的综合利用，支持能源技术和减排技术创新。加快发展生态农业，积极推广先进适用的生态农业技术，提高农业效益。发展生态产业、环保产业体系，促进再生资源加工行业的规范、快速发展。实施市区饮用水源改造工程，加强浔江、桂江等江河以及重点区域环境综合整治，保护青山绿水，争创全区生态文明示范城市。

　　强化节能减排工作 狠抓工业重点污染企业治污减排，进一步强化企业污染减排的主体责任，完善节能减排标准体系和主要污染物总量减排管理。严格执行以环境容量为基础的污染物总量控制制度，认真落实建设项目环境影响评价和“三同时”的要求，从源头上控制新污染源的产生。探索建立资源开采和生态环境治理恢复补偿机制，全面落实“一票否决制”，强化对污染源在线控制和监督性检测，提升环境监管水平，促进污染源全面达标排放。抓好重点耗能行业和重点耗能企业节能降耗，淘汰落后产能。

　　加快环保基础设施建设 推进市、县（市）污水垃圾处理项目及其配套管网、燃煤锅炉脱硫设施、重点污染源在线监控系统等环保基础设施建设，重点抓好市第二污水处理厂、梧州工业园区污水处理厂二期和赤水港工业园区污水处理厂等项目建设，力争上半年所有县（市）、园区的污水垃圾处理项目建成使用。

　　八、坚定不移为民办实事，让人民群众共享发展成果

　　今年继续实施为民办实事工程，筹措资金16.43亿元，办好以下12项实事：

　　（一）教育惠民工程 （1）筹措5000万元，改造存在安全隐患的中小学校舍4.5万平方米。（2）筹措225万元，继续资助中职学生学费。对进入中等职业教育学校学习的库区移民、扶贫异地安置家庭、城镇低保家庭、农村特困家庭的子女给予学费资助，平均每生每年资助1500元。（3）筹措1500万元，对中职学校特定学生发放助学金。（4）筹措75万元，继续资助中职学校特定专业学生第三学年生活费。（5）筹措3100万元，新建新兴小学，重建工厂路小学教学楼，扩建第二实验小学校区。

　　（二）医疗卫生保障惠民工程 （1）筹措4800万元，实施基本公共卫生保障工程，使年人均基本公共卫生服务经费标准达到15元，使城乡居民逐步享有均等化的基本公共卫生服务。（2）筹措500万元，实施艾滋病防治工程。（3）筹措500万元，实施母婴健康“一免二补”幸福工程。对符合条件办理结婚登记的人员及新婚夫妇实行免费婚前医学检查，对孕妇产前筛查及新生儿疾病筛查给予补助。（4）筹措375万元，改造5个乡镇级屠宰场，实施定点屠宰场病害猪无害化处理项目5个，确保上市生猪产品质量安全。（5）筹措1598万元，建造农村无害化卫生厕所2.5万座。

　　（三）社会保障惠民工程 （1）筹措3880万元，抓好苍梧县新型农村养老保险试点工作，实现全年农村居民参保人数达21万人以上。（2）筹措2.7亿元，继续完善新型农村合作医疗制度，补助标准从每人100元提高到120元，参合农民达225万人以上，参合率达90%以上，资金使用率达85%以上。（3）筹措2800万元，继续完善城镇居民基本医疗保险制度，补助水平从每人100元提高到120元，参保率达90%以上。（4）筹措720万元，建设60个行政村的五保供养服务设施，解决和改善农村五保户集中供养问题。（5）筹措1500万元，开展职业技能培训、创业培训和职业技能鉴定，通过小额担保贷款帮扶下岗失业人员、大学生、农村劳动者和退伍军人实现自主创业。

　　（四）文化广电惠民工程 （1）筹措150万元，建设6个村级公共服务中心。（2）筹措903万元，实施村村通广播电视工程，使778个20户以上的自然村通广播电视。（3）筹措672万元，建设16个乡镇综合文化站，建设业务用房面积5600平方米，并全部配置设备。

　　（五）安居工程 （1）筹措4500万元，改造3000户农村危房。（2）筹措7000万元，完成1000套廉租房三期工程，开工建设746套廉租房四期工程。（3）筹措7200万元，建设570套经济适用房。（4）筹措1.45亿元，完成枣冲15万平方米安置房建设。

　　（六）农村基础设施建设工程 （1）筹措5297万元，建设58个农村饮水安全工程。（2）筹措5213万元，新建四级路6条共48公里，改建三级路1条36公里。（3）筹措3122万元，新建非贫困村沼气池11800座、贫困村沼气池1147座。（4）筹措9280万元，实施11座病险水库除险加固工程。（5）筹措1000万元，实施第三批27个贫困村整村推进扶贫工程。

　　（七）城乡风貌改造工程 筹措3000万元，实施25个城乡风貌综合整治点的改造、小街小巷改造以及小绿地、小游园、小景点、小活动场所的建设。

　　（八）超级稻种子补贴项目工程 筹措600万元，全面推广超级稻及实施粮食高产配套工程。

　　（九）农村物流服务体系工程 筹措320万元，建设农资配送中心，完善农业生产资料流通体系。

　　（十）市区地质灾害整治工程 （1）筹措4.5亿元，推进平民冲、冰泉冲、石鼓冲地质灾害综合整治项目。（2）筹措1000万元，开展中山公园不稳定边坡、鸳江路桂江河岸岸坡等地质灾害隐患点治理。

　　（十一）珠山公园和狮卧山改造工程 筹措1500万元，将珠山公园改造成为历史博物公园，将狮卧山建设成为市民体育休闲公园。

　　（十二）农贸市场升级改造工程 筹措 500 万元，对市本级农贸市场进行升级改造，为群众创造良好的购物环境。

　　各位代表，为民办实事是政府的重要职责，我们一定全力把这些实事办好，让人民群众共享发展成果。

　　九、坚定不移维护社会稳定，营造良好发展环境

　　扎实开展平安梧州建设 贯彻落实自治区创建社会和谐稳定模范区的决策部署，扎实推进社会矛盾化解、社会管理创新、公正廉洁执法三项重点工作，健全社会矛盾纠纷排查调处和预警体系，继续开展“大排查、大走访、大调解”活动，切实解决影响社会稳定的突出问题。加强公安执法能力建设和社会治安综合治理，依法严厉打击违法犯罪活动。重视和加强社区建设，不断增加对社区的投入，充分发挥社区在平安创建中的作用。完善应急预警机制，有效预防和处置各类突发公共事件，加强自然灾害救助应急预案体系建设，推进救灾物资储备网络建设，切实维护社会和谐稳定，争创自治区社会和谐稳定模范市。

　　加强安全生产和食品药品监管 坚持“安全第一、预防为主、综合治理”的方针，按照“一岗双责”的要求，全面落实安全生产责任制和事故责任追究制，突出抓好矿山、危险化学品、道路交通等重点行业领域的安全生产，强化森林和公共场所火灾隐患的排查处理，加强安全生产应急救援能力建设，坚决遏制重特大安全事故的发生。大力开展产品质量和食品药品安全专项整治，强化源头监控和市场监管，确保产品质量和食品、药品安全。

　　开展和谐建设在基层活动 积极开展和谐单位、和谐乡镇、和谐村屯、和谐街道、和谐社区、和谐家庭、和谐学校、和谐企业、和谐邻里等建设活动，争创一批自治区级先进典型。

　　加强国防教育和支持部队建设 加强全民国防教育，继续做好国防动员和人民防空工作。支持驻梧部队、民兵预备役部队建设，落实军人优待抚恤政策，做好军队转业干部接收安置工作。深入开展“双拥共建”活动，巩固和发展军政军民团结。

　　十、坚定不移加快社会事业发展，提高政府公共服务水平

　　优先发展教育 实施中小学校舍安全工程，努力将学校建成安全最保障、家长最放心的地方。整合市区中小学教育资源，优化中小学校布局，重点抓好梧州高中等一批中小学校的新建和改扩建工作。加大职业教育攻坚力度，整合市本级公办职业教育资源，探索集团化办学模式。支持梧州学院、梧州职业学院等高等院校建设。全面落实教师绩效工资制度，提高教师待遇，稳定教师队伍，提高教育质量。

　　积极扩大就业 努力扩大就业和再就业，统筹做好大中专技校毕业生、农民工和就业困难群体特别是零就业家庭的就业工作，鼓励大学毕业生到基层、企业就业和自主创业。做好返乡农民工和农村劳动力转移就业工作，促进农民工就地就近就业，努力实现农村劳动力转移新增就业5.5万人。加强城乡劳动力职业技能培训，提高劳动者的职业技能水平和就业能力。

　　完善社会保障体系 全面推进统筹城乡社会养老保险制度，进一步扩大覆盖面。积极探索医疗保险市级统筹，完善城乡居民合作医疗保险制度与城镇职工基本医疗保险制度，妥善解决国有困难企业和集体困难企业退休职工医疗保险问题。进一步加大失业、工伤、生育等社会保险扩面力度。加强低收入群众的帮扶救助工作，完善城乡居民最低生活保障制度。

　　加强人口和计划生育工作 严格落实目标管理责任制，重点加强农村人口和流动人口计生服务管理，推进人口计生体制机制创新，抓好“诚信计生”试点，推行婚检“一站式”服务，提高人口计划生育覆盖率和服务水平。深入开展农村计划生育家庭就业创业援助行动和农村困难计划生育家庭帮扶活动，大力实施新农村新家庭计划，推进“婚育新风进万家”活动和关爱女孩行动，综合治理出生人口性别比偏高问题，稳定低生育水平，提高出生人口素质。

　　加快发展卫生事业 加强城乡卫生基础设施建设，新建市人民医院，扩建市第二人民医院，完成红会医院综合楼建设。健全县、乡、村三级农村医疗卫生服务网络。落实重大公共卫生服务项目，实施乙肝疫苗补种、贫困白内障患者复明、农村妇女健康检查等工程。加强重大传染病的预防控制。加大扶持中医药发展力度，创建独具特色的中医医疗、保健、科研体系，提高综合服务能力。积极推进医药卫生体制改革，提高基本医疗保障覆盖面，促进基本公共卫生服务逐步均等化，努力实现人人享有基本医疗服务的目标。

　　抓好其他各项事业 大力开展全民健身活动，加快市体育中心建设。加强公务员和专业技术人员的管理，继续推进事业单位改革。加大扶贫工作力度，积极实施第三批整村推进扶贫。强化国资监管、统计、审计、档案、编制、法制工作。加强对台、外事、侨务、民族、宗教工作。协调发展工会、共青团、妇女儿童、老龄事业，支持发展红十字人道和慈善事业。充分发挥地震、气象、水文等工作在防灾减灾中的作用。

　　组织力量编制好“十二五”规划纲要及相关专项规划，科学谋划梧州今后五年的发展目标和重大战略任务。做好第六次人口普查工作。

　　各位代表！挑战前所未有，机遇前所未有，希望前所未有。面对全市人民的重托，我们必须进一步增强贯彻落实科学发展观的自觉性和坚定性，进一步增强加快发展的责任感和使命感，进一步转变政府职能，提高社会管理和公共服务能力。一是加强行政效能建设。严格执行首问负责制、限时办结制、责任追究制，继续推进行政审批制度改革，优化审批流程，精简审批环节，缩短审批时限，努力把政务服务中心建设成为高效、便民、廉洁的“一站式”服务平台。二是加强作风建设。始终保持奋发有为、只争朝夕的工作干劲，倡导简政、高效、务实的作风，坚决克服官僚主义、形式主义，戒空、戒虚、戒假、戒骄、戒懒、戒奢。三是加强民主法制建设。自觉接受人大监督和政协民主监督，认真听取民主党派、无党派人士和各人民团体的意见，主动接受新闻舆论和社会公众监督。大力推行政务公开，健全政府信息发布制度，提高政府工作透明度。全面推进依法行政，健全和执行重大决策的规则和程序，切实做到依法决策、科学决策、民主决策。四是加强廉政建设。全面贯彻落实党风廉政建设各项规定，加强对领导干部、人财物管理使用、关键岗位的监督和制约。加强经济责任审计，强化行政监督，坚决查处违法违纪案件，以清正廉洁取信于民。继续深入开展专项治理，纠正部门和行业的不正之风，坚决维护人民群众的切身利益。

　　各位代表，站在新起点，谋求新发展，实现新突破，是全市人民的共同期待，是我们的光荣使命和神圣职责。让我们紧密团结在以胡锦涛同志为总书记的党中央周围，高举中国特色社会主义伟大旗帜，深入贯彻落实科学发展观，万众一心，开拓进取，奋发有为，为开创我市科学发展、和谐发展、跨越发展新局面，夺取全面建设小康社会新胜利，谱写人民美好生活新篇章而努力奋斗！
